# Interpretation of multi-detector computed tomography images before dissection may allow detection of vascular anomalies: a postmortem study of anomalous origin of the right subclavian artery and the right vertebral artery

**DOI:** 10.1007/s12565-012-0151-0

**Published:** 2012-08-29

**Authors:** Noboru Sakamoto, Hidenobu Miyaso, Masatoshi Komiyama, Yota Sugata, Takane Suzuki, Toshihiko Kohno, Hirotaro Iwase, Mutsumi Hayakawa, Go Inokuchi, Chisato Mori, Yoshiharu Matsuno

**Affiliations:** 1 Department of Bioenvironmental Medicine, Graduate School of Medicine, Chiba University, Inohana 1-8-1, Chuo-ku, Chiba, 260-8670 Japan; 2 Department of Legal Medicine, Graduate School of Medicine, Chiba University, Chiba, Japan

**Keywords:** Computed tomography, Variation, Subclavian artery, Vertebral artery, Anatomical education

## Abstract

The Graduate School of Medicine at Chiba University is planning to introduce computed tomography (CT) images of donated cadavers to the gross anatomy laboratory. Here we describe an anomaly of the right subclavian artery that was detected by interpretation of CT images prior to dissection. The anomaly was verified to be the right subclavian artery, as the last branch of the aortic arch, by subsequent dissection of the cadaver. We also identified an anomalous origin of the right vertebral artery by dissection. This anomaly was also visible on CT images, although it had not been recognized in the first interpretation of the CT images. Our results suggest that branching anomalies of arteries with a diameter of >1 cm are detectable on CT images even without the injection of contrast medium. We also discuss the utility of interpreting CT images prior to dissection as a means by which medical students can gain a better understanding of human body during the gross anatomy laboratory.

## Introduction

In recent years the interpretation of computed tomography (CT) images has become indispensable for the diagnosis of diseases and the evaluation of treatment in the clinical setting. It is also possible that anatomical anomalies in patients may also be identified when CT images are being interpreted (Goray et al. 2005; Ka-Tak et al. 2007; Park et al. 2008). It has also been reported that partial resection of the pectoralis major muscle in association with surgery for breast cancer and anomalous shape of the vertebral column with the lateral curvature can be recognized prior to dissection based on the interpretation of CT images of cadavers (Matsuno et al. 2009). Thus, interpretation of CT images may be helpful not only in the clinical setting but also in gross anatomy laboratory. In fact, the use of CT images in anatomical education classes has already been initiated in Japan (Murakami et al. 2011). The Graduate School of Medicine, Chiba University, is also planning to introduce CT images of donated cadavers to its gross anatomy laboratory. As such, medical students can dissect cadavers after viewing CT images of their dissecting cadavers that were obtained in advance.

Here, we describe a cadaver in which we detected an anomaly of the right subclavian artery by interpreting CT images prior to dissection. We verified the anomaly by subsequent dissection of the cadaver and also identified an anomalous origin of the right vertebral artery that had not been recognized on the CT images. We also discuss the utility of interpreting CT images before dissection for identifying vascular anomalies and for gaining a better understanding of the human body by students in the gross anatomy laboratory.

## Methods

### Conducting CT scans of cadavers

At Chiba University, consent for cadaver use for the purpose of medical education, anatomy studies, and CT scans is obtained from members of the whole body donation registry and their families. CT scans are performed on cadavers only after such consent has been obtained. All CT scans of cadavers have been approved by the ethics committee of Graduate School of Medicine, Chiba University (No. 521).

### CT scan of the cadaver and settings for CT image interpretation

The cadaver used in this study was an 84-year-old Japanese woman who died of lung cancer. After the cadaver had been embalmed and fixed by perfusion with a solution containing formaldehyde, a CT scan was performed. No contrast medium was injected.

The whole cadaver was scanned at a 1.25-mm slice interval using an ECLOS 16-line Multi-detector Computed Tomography (Hitachi Medical Co., Tokyo, Japan). Scanning parameters were 120 kV and 200 mA for the tube voltage, and 1.25 × 16 mm for the beam collimation. CT images were reconstructed at a section thickness of 1.25 mm. The software used to view CT image data was OsiriX MD ver. 1.0 64-bit.

## Results

### Interpretation of CT images before dissection

**Fig. 1:**
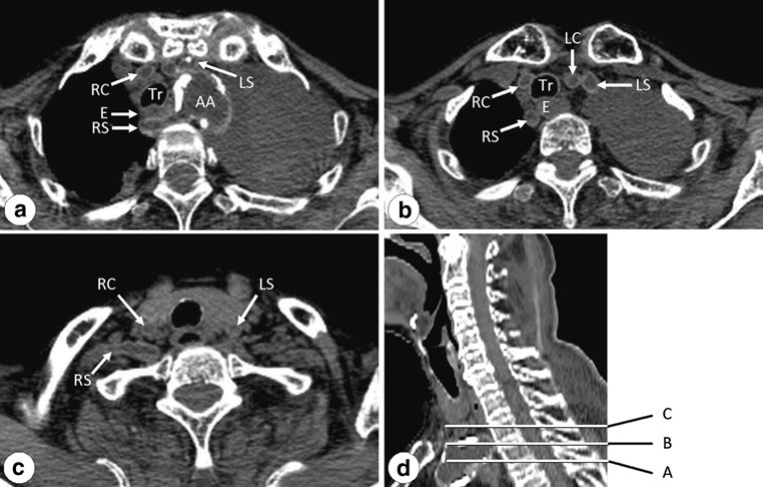
Computed tomograph images of horizontal sections at the level of the third thoracic vertebra (**a**), at the boundary between the second and third thoracic vertebrae (**b**), and at the second thoracic vertebra (**c**). **d** A sagittal section indicating the horizontal section levels of CT images in** a**,** b**, and** c** (*A*,* B*, and* C*, respectively). The right subclavian artery can be confirmed to be crossing between the vertebral body and esophagus (**a**). *AA* Aortic arch, *E* esophagus, *LC* left common carotid artery, *LS* left subclavian artery, *RC* right common carotid artery, *RS* right subclavian artery, *Tr* trachea

Interpretation of the CT images before dissection revealed a branching anomaly of the aortic arch (Fig. 1a–d). In a horizontal section at the level of the third thoracic vertebra, an unusual structure that appeared to be an artery was detected passing behind the esophagus (Fig. 1a). This structure seemed to branch from the aortic arch. When it was followed upward, it was found to be located on the right side of the esophagus at the level of the boundary between the second and third thoracic vertebrae (Fig. 1b) and then turned to the right at the level of the second thoracic vertebra (Fig. 1c). Three arteries were observed in front of the trachea (Fig. 1a, b). One of these ascended along the right side of the trachea without branching off the right subclavian artery (Fig. 1b, c) and thus appeared not to be the brachiocephalic artery, but the right common carotid artery. The other two on the left side of the trachea ascended taking normal courses and seemed to be the left common carotid artery and the left subclavian artery, respectively (Fig. 1b, c). Thus, the artery-like structure passing behind the esophagus seemed to be the right subclavian artery.

### Assessment based on dissection of the area of malformation detected on CT images

**Fig. 2:**
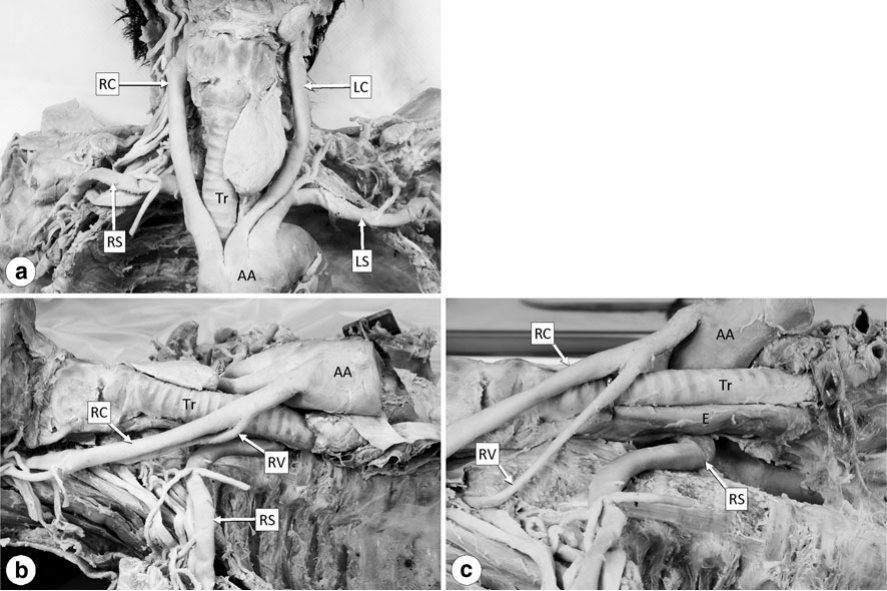
Photographs showing the anterior (**a**) and right side view (**b**, **c**) of the arteries branching from the aortic arch (*AA*). **c** The AA is pulled towards the front to show the right vertebral artery (*RV*) and esophagus (*E*). All abbreviations are as given in Fig. 1

**Fig. 3:**
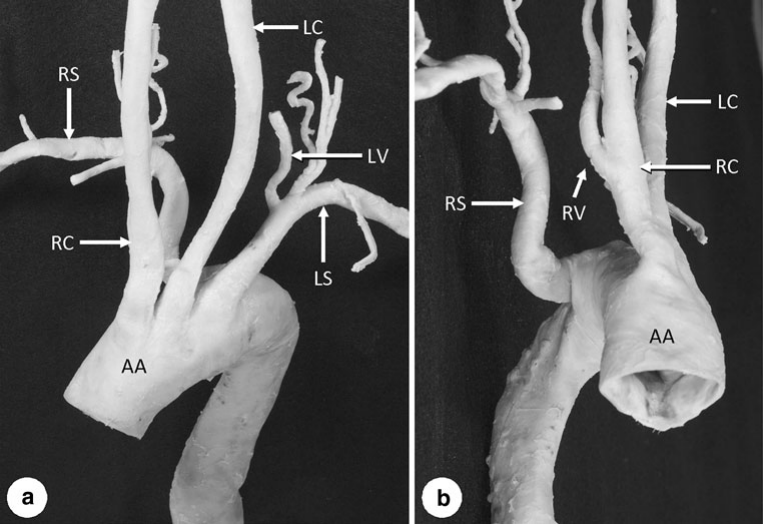
Photographs showing the anterior (**a**) and right side view (**b**) of the aortic arch (*AA*) and its branches. All abbreviations are as given in Fig. 1

The dissection conducted to assess the branching anomaly of the aortic arch detected on the CT images confirmed malformation of the right subclavian artery, i.e. as the last branch of the aortic arch (Figs. 2, 3). The right subclavian artery branched from the medial posterior surface of the aortic arch as its last branch at the level of the second and third thoracic vertebra (Fig. 3). It coursed obliquely to the upper right between the posterior surface of the esophagus and the anterior surface of the second and third thoracic vertebral bodies and then ascended (Fig. 2b, c) and crossed rightward to become the axillary artery at the level of the first thoracic vertebra (Fig. 2a).

The right inferior laryngeal nerve branched directly from the right vagus nerve in the cervical region, and thus the right recurrent laryngeal nerve was absent. In contrast, the left recurrent laryngeal nerve was normal. The ligamentum arteriosum was also normal.

In addition to the above-described anomalous origin of the right subclavian artery, it was also revealed that the right vertebral artery branched from the right common carotid artery and entered the transverse foramen of the fifth cervical vertebra (Figs. 2b, c, 3b). In contrast, the left vertebral artery branched from the left subclavian artery (Fig. 3a) and ascended through the transverse foramen from the sixth to the first cervical vertebra, thus showing the normal form.

Table 1 shows the diameters (major axis/minor axis) of the arteries branching from the aortic arch. The diameters were measured just after branching (at approximately 5 mm from the branching site). The major axis of the right subclavian artery had a diameter of 13.2 mm and its minor axis a diameter of 4.5 mm; The right subclavian artery also had a flattened appearance from its origin up to the location on the right side of the esophagus (Fig. 3b). This appearance might have been caused by compression because the right subclavian artery coursed obliquely to the upper right between the esophagus and thoracic vertebral bodies. However, it is unclear whether the compression occurred during lifetime or after death. The esophagus showed only slight compression, possibly due to the right subclavian artery, and was also flat (Fig. 2c).

**Table 1:** Diameters (major axis/minor axis) of the arteries branching from the aortic arch

Arteries	Major axis (mm)	Minor axis (mm)
Subclavian artery		
Right	13.2^a^	4.5
Left	11.0	7.8
Common carotid artery		
Right	11.7	7.9
Left	9.9	7.9
Vertebral artery		
Right	5.5	4.6
Left	4.5	3.6
Aortic arch		
Proximal to the branching site of the right subclavian artery	32.0	27.0
Distal to the branching site of the right subclavian artery	33.2	23.3

^a^Each diameter was measured at approximately 5 mm from the branching site

The major axis of the right vertebral artery had a diameter of 5.5 mm and the minor axis a diameter of 4.6 mm (Table 1). Both diameters were larger than those of the left vertebral artery (major axis 4.5 mm, minor axis 3.6 mm).

### Review of CT images after dissection

**Fig. 4:**
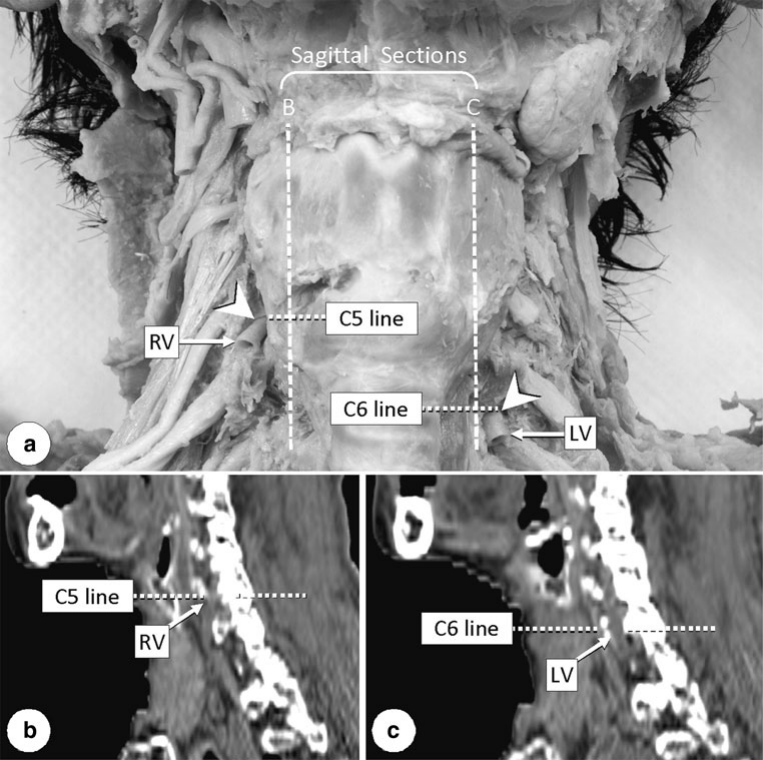
Anterior view (**a**) and sagittal sections (**b**, **c**) showing vertebral arteries entering the transverse foramina. *Arrowheads* (**a**) indicate the entering sites.* Vertical lines* (*B* and* C*) in **a** indicate the positions of sagittal sections in **b** and **c**, respectively. *C5* Fifth cervical vertebra, *C6* sixth cervical vertebra,* LV* left vertebral artery,* RV* right vertebral artery

Although the interpretation of CT images before dissection revealed the aberrant right subclavian artery, the anomaly of the right vertebral artery was not identified at this time. When the anomalous origin and course of the right vertebral artery detected by dissection were checked again on the CT images, it could be confirmed on the horizontal and sagittal section images at the level of the fifth cervical vertebra that this artery enters the right transverse foramen of the fifth cervical vertebra (Fig. 4). However, the branching site from the right common carotid artery could not be identified due to insufficient image clarity.

### What organs can be interpreted on CT images without contrast medium?

Even without contrast medium, bones and calcified deposits along arteries were easy to interpret on the CT images of the cadaver. Also, major organs (heart, lung, liver, kidney, brain, spinal cord, major muscles like biceps brachii muscle and gluteus maximus muscle, etc.) were recognizable, but the interpretation of veins, peripheral nerves, and smaller muscles were difficult.

## Discussion

### Anomalous origin of the right subclavian artery and the right vertebral artery

The branching pattern of the aortic arch in the cadaver under discussion resembled that of Type G or Type CG of the Adachi–Williams–Nakagawa classification (Adachi 1928; Williams et al. 1932; Nakagawa 1939), in which both types have a retroesophageal right subclavian artery and anomalous origin of the vertebral arteries. However, the left vertebral artery in our study was normal, although anomalous origin of the left vertebral artery is also involved in both Type G and Type CG. Thus, classification of the branching pattern is slightly difficult in this cadaver.

Normally, the right subclavian artery is formed with the seventh intersegmental artery, which arises from the right dorsal aorta, after obliteration of the peripheral segment of the right dorsal aorta (Moore 1982; Wasserman et al. 1992; Lemke et al. 1999). If the obliteration occurs proximally to the seventh intersegmental artery, the right subclavian artery starts to branch from the aortic arch as its last branch (Wasserman et al. 1992; Lemke et al. 1999). It has been reported that the right recurrent laryngeal nerve is usually absent when the right subclavian artery branches from the aortic arch and takes a retroesophageal course (Kawashima and Sasaki 2005). In our study, the right recurrent laryngeal nerve was also absent from the cadaver. The vertebral artery is formed by the development of longitudinal anastomoses between the cervical (from the first to the seventh) intersegmental arteries, and thus the vertebral artery normally arises from the subclavian artery and enters the transverse foramen of the sixth cervical vertebra (Lemke et al. 1999; Ikegami et al. 2007; Park et al. 2008). In our study, the segment of the right dorsal aorta and the longitudinal anastmosis between the sixth and seventh intersegmental arteries might have regressed and, instead, the peripheral segment of the right dorsal aorta and the proximal part of the sixth intersegmental artery might have survived. If this were to be the case, the proximal part of the right vertebral artery in this cadaver was formed with the fourth aortic arch, a part of the dorsal aorta and proximal part of the sixth intersegmental artery. This may be related with the fact that the diameter of the right vertebral artery is larger than that of the left vertebral artery in this cadaver.

The incidence of the right subclavian artery as the last branch of the aortic arch has been reported in Japanese adults as ranging from 0.15 to 1.6 % with an average of about 0.5 % (Komiyama et al. 1995). With respect to the vertebral artery, the left one has been reported to show anomalous origin at a frequency of 2.4–5.9 % in several large autopsy series (Lemke et al. 1999; Akdeniz et al. 2007; Ikegami et al. 2007). However, an anomalous origin of the right vertebral artery has been reported to be rarer, being detected as a coincidental finding during surgery or at autopsy (Akdeniz et al. 2007). In fact, many of the reports describing anomalous origin of the right vertebral artery are coincidental findings during surgery (Palmer 1977; Rieger and Huber 1983; Chen et al. 1998; Lemke et al. 1999; Best and Bumpers 2002; Yanik et al. 2004; Goray et al. 2005; Akdeniz et al. 2007; Ka-Tak et al. 2007; Satti et al. 2007; Kau et al. 2008; Park et al. 2008; Kim et al. 2009), although such findings at autopsy have also been reported (Mori et al. 1990; Gluncic et al. 1999; Fazan et al. 2004; Ikegami et al. 2007). The same concomitant anomalies of the right subclavian artery and right vertebral artery as those observed in our study have been reported previously, although the vertebral artery enters the transverse foramen at various levels (Mori et al. 1990; Gluncic et al. 1999; Fazan et al. 2004; Ka-Tak et al. 2007; Kau et al. 2008; Park et al. 2008; Kim et al. 2009). Consequently, the findings we report here may be not be that exceptional.

### Possibility of detecting anomalies on CT images before dissection

We detected branching anomaly of the subclavian artery on CT images examined prior to dissection. At that time, however, anomaly of the vertebral artery could not be detected. The detection—or not—of these anomalies may depend on the size and thickness of the wall of arteries, which affect the visibility of arteries on CT images. The subclavian artery is approximately 1 cm in diameter and considered to be an elastic type artery which has a thicker wall. In comparison, the vertebral artery is usually 4–5 mm in diameter and might be a muscular type artery with a thinner wall. Our results suggest that branching anomalies of arteries with a diameter of >1 cm are detectable on CT images even without the injection of contrast medium. The diagnosis of anomalies of thinner arteries that are several millimeters in diameter may also be possible depending on the techniques applied to interpret the CT images. The use of contrast medium definitely makes the diagnosis easier, as has been shown in previous reports (Palmer 1977; Rieger and Huber 1983; Chen et al. 1998; Lemke et al. 1999; Best and Bumpers 2002; Yanik et al. 2004; Goray et al. 2005; Akdeniz et al. 2007; Ka-Tak et al. 2007; Satti et al. 2007; Kau et al. 2008; Park et al. 2008; Kim et al. 2009; Matsuno et al. 2009).

Various anomalies are usually encountered in the gross anatomy laboratory. However, we believe that there may be numerous cadavers in which these anomalies go undetected in the gross anatomy laboratory because medical students who are just learning normal structure are not yet skilled in differentiating anomalies from normal structure. If the presence of an anomaly is known prior to dissection, students can pay attention to the anomaly and dissect the cadaver carefully to find the anomaly. The interpretation of CT images prior to dissection may therefore be a useful educational tool for medical students to detect anomalies during the gross anatomy laboratory.

### For a better understanding of human body structure by medical students

Computed tomography images are frequently used in the clinical setting for the diagnosis of diseases and evaluation of treatments. In order to interpret CT images, it is important for the physician to be able to correlate two-dimensional CT images with the actual three-dimensional structures of the body. Although medical students can observe inner structures of the human body three-dimensionally in the gross anatomy laboratory, they sometimes lack knowledge of the two-dimensional aspects of the structures during the actual dissection. Clinical trainees also have few opportunities to relearn inner structures of the whole body three-dimensionally by dissection, even though they have many opportunities to view two-dimensional CT images. Thus, when dissecting a cadaver, medical students may find it easier to correlate CT images with the real structure if they have had the opportunity to view CT images of that cadaver prior to the dissection. Thus, CT scanning of donated cadavers and the introduction of CT images of the cadavers to gross anatomy laboratories may be a very useful educational approach that provides medical students with a better understanding of human body structure. If clear three-dimensional reconstructed images can be obtained from cadavers, such images would clearly be useful for anatomical education. However, it is difficult to obtain clear three-dimensional images at the present time due to insufficient contrast between organs. Thus, improved methods that allow optimal imaging of CT specimens are urgently needed.
